# Barium bis­[tetra­fluorido­bromate(III)]

**DOI:** 10.1107/S2414314621007355

**Published:** 2021-07-20

**Authors:** Sergei I. Ivlev, Florian Kraus

**Affiliations:** a Philipps-Universität Marburg, Fachbereich Chemie, Hans-Meerwein-Str. 4, 35032 Marburg, Germany; Vienna University of Technology, Austria

**Keywords:** crystal structure, barium, tetra­fluorido­bromate(III), re-refinement

## Abstract

Single crystals of barium bis­[tetra­fluorido­bromate(III)], Ba[BrF_4_]_2_, were obtained in the form of tiny blocks. Crystal-structure refinement of Ba[BrF_4_]_2_ from single-crystal X-ray diffraction data confirmed the previous model obtained on the basis of powder data [Ivlev *et al.* (2014 ▸). *Eur. J. Inorg. Chem*. pp. 6261–6267], but with all atoms refined with anisotropic displacement parameters. The crystal structure consists of two symmetry-independent barium cations that are each coordinated by twelve fluorine atoms, forming edge-sharing polyhedra, and an almost square-planar [BrF_4_]^−^ anion. The compound crystallizes in the Ba[AuF_4_]_2_ structure type.

## Abstract 2

The crystal structure of Ba[BrF_4_]_2_ was refined against single-crystal X-ray diffraction data collected at 100 K, confirming the previous model from powder data.

## Graphical abstract

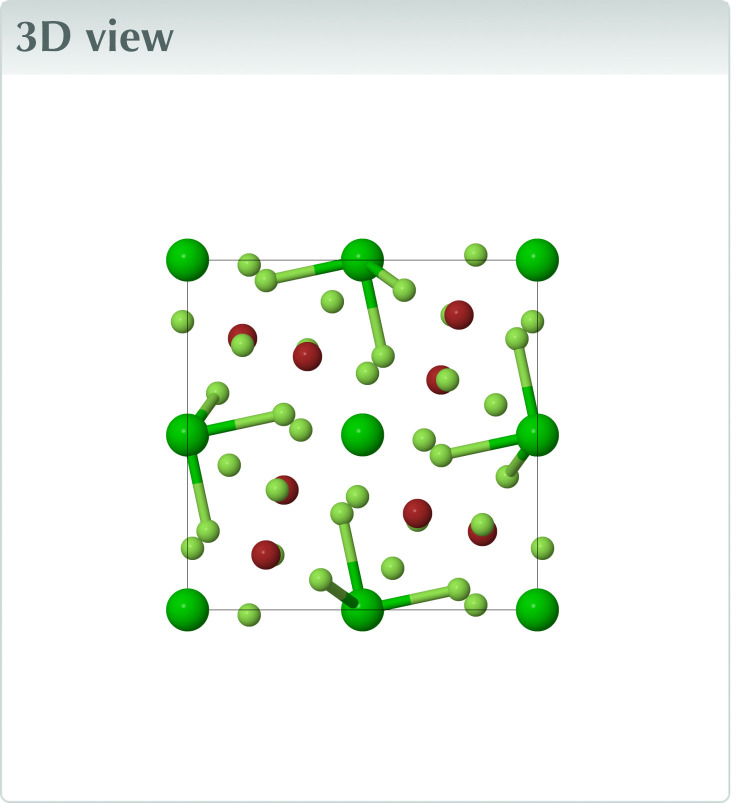

## Structure description

The first synthesis of Ba[BrF_4_]_2_ was performed by Sharpe & Emeléus (1948 ▸) by treating anhydrous barium chloride or barium fluoride with bromine trifluoride. The product was, however, only characterized by means of a qu­anti­tative elemental analysis. The thermal properties of Ba[BrF_4_]_2_ were later studied by Kiselev and co-workers, who investigated the thermal decomposition of Ba[BrF_4_]_2_ to yield barium fluoride (Kiselev *et al.*, 1987 ▸). To the best of our knowledge, our report on the crystal structure of Ba[BrF_4_]_2_ determined from X-ray and neutron powder diffraction data at 300 K was the first structural investigation of the title compound (Ivlev *et al.*, 2014 ▸). We showed that Ba[BrF_4_]_2_ crystallizes in the space group *I* 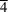 and adopts the Ba[AuF_4_]_2_ structure type. Here we present our results on the re-refinement of the crystal structure of Ba[BrF_4_]_2_ from single-crystal X-ray diffraction data at 100 K.

**Figure 1:**
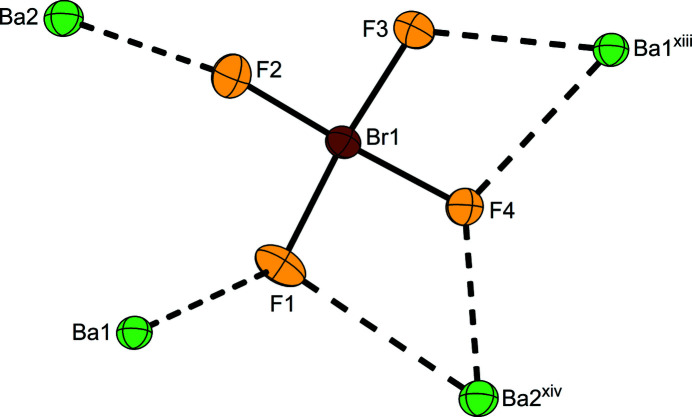
The closest contacts between one [BrF_4_]^−^ anion and surrounding Ba^2+^ cations. Displacement ellipsoids are shown at the 50% probability level. [Symmetry codes: (xiii) *x* + 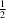 , *y* + 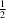 , *z* + 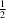 ; (xiv) *x* + 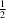 , *y* − 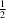 , *z* + 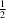 .]

**Figure 2:**
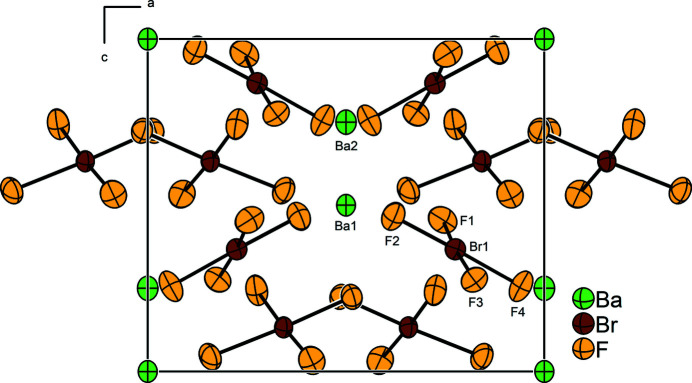
The crystal structure of Ba[BrF_4_]_2_ in a projection along the *b* axis. Displacement ellipsoids are shown at the 50% probability level.

As expected, the unit-cell parameters of the single-crystal study at 100 K (Table 1 ▸) are smaller than those determined during the powder study at 300 K, *a* = 9.65081 (11), *c* = 8.03453 (13) Å, *V* = 748.32 (2) Å^3^ (Ivlev *et al.*, 2014 ▸). The crystal structure contains two symmetry-independent Ba^2+^ cations on special Wyckoff positions 2*a* (site symmetry 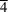 ..) and 2*d* ( 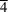 ..), respectively. Each Ba site is coordinated by twelve F atoms forming edge-sharing polyhedra. The Ba⋯F distances lie in the range of 2.680 (14)–3.324 (16) Å [powder data at RT yielded the range of 2.696 (3)–3.376 (3) Å]. The bromine atom occupies the general Wyckoff position 8*g* and is coordinated by four fluorine atoms also located on general positions in an almost square-planar shape. The resulting Br—F bond lengths are 1.829 (13), 1.861 (12), 1.934 (13), and 1.935 (13) Å, which is comparable with our previous model on basis of powder data [*cf.*: 1.800 (4), 1.856 (4), 1.902 (4), 1.935 (2) Å]. The two longer Br—F bond lengths correspond to the F atoms coordinating two barium cations each. The two other fluorine atoms coordinate only to one barium cation each and thus have shorter Br—F bond lengths. The F—Br—F *cis*-angles are 84.9 (6), 89.6 (6), 92.6 (6) and 92.9 (6)°, which corresponds with the previously published results: 85.14 (16), 90.02 (13), 91.80 (15), 93.04 (18)°. Fig. 1 ▸ shows the closest contacts between one [BrF_4_]^−^ anion and its surrounding Ba^2+^ cations, and Fig. 2 ▸ shows the packing of the cations and anions in the crystal structure.

## Synthesis and crystallization

Tiny crystals of barium tetra­fluorido­bromate(III) were obtained by direct reaction of bromine trifluoride with barium fluoride in a closed Teflon vessel. In contrast to Rb[BrF_4_] (Malin *et al.*, 2019 ▸) and Cs[BrF_4_] (Malin *et al.*, 2020 ▸), it was not possible to improve the crystal quality by melting and recrystallization since Ba[BrF_4_]_2_ decomposes before reaching its melting point.

## Refinement

Crystal data, data collection and structure refinement details are summarized in Table 1 ▸. Because of very small size of the crystals, we had to employ a diffractometer with a Cu source to improve the reflection intensities at the cost of a more complicated absorption correction.

## Supplementary Material

Crystal structure: contains datablock(s) I. DOI: 10.1107/S2414314621007355/wm4148sup1.cif

Structure factors: contains datablock(s) I. DOI: 10.1107/S2414314621007355/wm4148Isup2.hkl

pdf file with requested changes and comments. DOI: 10.1107/S2414314621007355/wm4148sup3.pdf

CCDC reference: 2096684

Additional supporting information:  crystallographic information; 3D view; checkCIF report

## Figures and Tables

**Table 1:** Experimental details

Crystal data	
Chemical formula	Ba[BrF_4_]_2_
*M*_r_	449.16
Crystal system, space group	Tetragonal, *I* 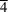
Temperature (K)	100
*a*, *c* (Å)	9.5823 (6), 8.0380 (11)
*V* (Å^3^)	738.05 (14)
*Z*	4
Radiation type	Cu *K*α
μ (mm^−1^)	55.60
Crystal size (mm)	0.02 × 0.02 × 0.01

Data collection	
Diffractometer	Stoe Stadivari
Absorption correction	Multi-scan [*X-AREA* (Stoe, 2020 ▸) based on Koziskova *et al.*, (2016 ▸)]
*T*_min_, *T*_max_	0.068, 0.362
No. of measured, independent and observed [*I* > 2σ(*I*)] reflections	2012, 746, 688
*R*_int_	0.038
(sin θ/λ)_max_ (Å^−1^)	0.637

Refinement	
*R*[*F* ^2^ > 2σ(*F* ^2^)], *wR*(*F* ^2^), *S*	0.049, 0.127, 1.08
No. of reflections	746
No. of parameters	50
Δρ_max_, Δρ_min_ (e Å^−3^)	1.84, −1.05
Absolute structure	Flack *x* determined using 266 quotients [(*I* ^+^)−(*I* ^−^)]/[(*I* ^+^)+(*I* ^−^)] (Parsons *et al.*, 2013 ▸)
Absolute structure parameter	−0.012 (17)

Computer programs: *X-AREA* (Stoe, 2020 ▸), *SHELXT* (Sheldrick, 2015*a* ▸), *SHELXL* (Sheldrick, 2015*b* ▸), *DIAMOND* (Brandenburg, 2020 ▸) and *publCIF* (Westrip, 2010 ▸).

## full crystallographic data

### Crystal data

**Table:**

Ba^2+^·2BrF_4_^−^	*D*_x_ = 4.042 Mg m^−^^3^
*M**_r_* = 449.16	Cu *K*α radiation, λ = 1.54186 Å
Tetragonal, *I*4	Cell parameters from 2706 reflections
*a* = 9.5823 (6) Å	θ = 6.5–79.3°
*c* = 8.0380 (11) Å	µ = 55.60 mm^−^^1^
*V* = 738.05 (14) Å^3^	*T* = 100 K
*Z* = 4	Block, colorless
*F*(000) = 792	0.02 × 0.02 × 0.01 mm

### Data collection

**Table:**

Stoe Stadivari diffractometer	746 independent reflections
Radiation source: GeniX 3D HF Cu	688 reflections with *I* > 2σ(*I*)
Graded multilayer mirror monochromator	*R*_int_ = 0.038
Detector resolution: 5.81 pixels mm^-1^	θ_max_ = 79.1°, θ_min_ = 6.5°
rotation method, ω scans	*h* = −11→12
Absorption correction: multi-scan [X-AREA (Stoe, 2020) based on Koziskova *et al.*, (2016)]	*k* = −10→6
*T*_min_ = 0.068, *T*_max_ = 0.362	*l* = −10→9
2012 measured reflections	

### Refinement

**Table:**

Refinement on *F*^2^	Primary atom site location: structure-invariant direct methods
Least-squares matrix: full	*w* = 1/[σ^2^(*F*_o_^2^) + (0.091*P*)^2^ + 5.3796*P*] where *P* = (*F*_o_^2^ + 2*F*_c_^2^)/3
*R*[*F*^2^ > 2σ(*F*^2^)] = 0.049	(Δ/σ)_max_ < 0.001
*wR*(*F*^2^) = 0.127	Δρ_max_ = 1.84 e Å^−^^3^
*S* = 1.08	Δρ_min_ = −1.05 e Å^−^^3^
746 reflections	Absolute structure: Flack *x* determined using 266 quotients [(*I*^+^)-(*I*^-^)]/[(*I*^+^)+(*I*^-^)] (Parsons *et al.*, 2013)
50 parameters	Absolute structure parameter: −0.012 (17)
0 restraints	

### Special details

**Table:**

Geometry. All esds (except the esd in the dihedral angle between two l.s. planes) are estimated using the full covariance matrix. The cell esds are taken into account individually in the estimation of esds in distances, angles and torsion angles; correlations between esds in cell parameters are only used when they are defined by crystal symmetry. An approximate (isotropic) treatment of cell esds is used for estimating esds involving l.s. planes.

### Fractional atomic coordinates and isotropic or equivalent isotropic displacement parameters (Å^2^)

**Table:**

	*x*	*y*	*z*	*U*_iso_*/*U*_eq_	
Ba1	0.500000	0.500000	0.500000	0.0243 (6)	
Br1	0.77576 (19)	0.84257 (18)	0.6325 (2)	0.0238 (5)	
F1	0.7435 (16)	0.6574 (14)	0.5444 (17)	0.041 (3)	
Ba2	0.500000	1.000000	0.250000	0.0293 (6)	
F2	0.6192 (14)	0.9136 (15)	0.5334 (19)	0.041 (3)	
F3	0.8236 (13)	1.0143 (13)	0.7242 (17)	0.036 (3)	
F4	0.9413 (13)	0.7748 (13)	0.744 (2)	0.040 (3)	

### Atomic displacement parameters (Å^2^)

**Table:**

	*U*^11^	*U*^22^	*U*^33^	*U*^12^	*U*^13^	*U*^23^
Ba1	0.0219 (7)	0.0219 (7)	0.0291 (12)	0.000	0.000	0.000
Br1	0.0251 (10)	0.0197 (9)	0.0267 (9)	−0.0021 (7)	−0.0004 (8)	0.0000 (7)
F1	0.050 (8)	0.030 (7)	0.043 (7)	−0.013 (6)	0.008 (6)	−0.008 (5)
Ba2	0.0257 (8)	0.0257 (8)	0.0364 (13)	0.000	0.000	0.000
F2	0.033 (7)	0.042 (7)	0.048 (8)	0.004 (6)	−0.009 (6)	−0.004 (6)
F3	0.042 (7)	0.027 (6)	0.038 (8)	−0.001 (5)	−0.006 (6)	−0.003 (5)
F4	0.034 (7)	0.029 (6)	0.057 (8)	0.003 (6)	−0.017 (7)	−0.006 (6)

### Geometric parameters (Å, º)

**Table:**

Ba1—F3^i^	2.791 (13)	Br1—F2	1.829 (13)
Ba1—F3^ii^	2.791 (13)	Br1—F3	1.861 (12)
Ba1—F3^iii^	2.791 (13)	Br1—F4	1.934 (13)
Ba1—F3^iv^	2.791 (13)	Br1—F1	1.935 (13)
Ba1—F1^v^	2.801 (14)	Ba2—F2^viii^	2.680 (14)
Ba1—F1^vi^	2.801 (14)	Ba2—F2^ix^	2.680 (14)
Ba1—F1^vii^	2.801 (14)	Ba2—F2^x^	2.680 (14)
Ba1—F1	2.801 (14)	Ba2—F2	2.680 (14)
Ba1—F4^ii^	3.034 (14)	Ba2—F4^xi^	2.693 (12)
Ba1—F4^iv^	3.034 (14)	Ba2—F4^vii^	2.693 (12)
Ba1—F4^iii^	3.034 (14)	Ba2—F4^iii^	2.693 (12)
Ba1—F4^i^	3.034 (14)	Ba2—F4^xii^	2.693 (12)
			
F3^i^—Ba1—F3^ii^	129.1 (3)	F4^ii^—Ba1—F4^iii^	94.6 (6)
F3^i^—Ba1—F3^iii^	129.1 (3)	F4^iv^—Ba1—F4^iii^	117.4 (3)
F3^ii^—Ba1—F3^iii^	74.9 (6)	F3^i^—Ba1—F4^i^	52.0 (4)
F3^i^—Ba1—F3^iv^	74.9 (6)	F3^ii^—Ba1—F4^i^	95.7 (4)
F3^ii^—Ba1—F3^iv^	129.1 (3)	F3^iii^—Ba1—F4^i^	168.1 (4)
F3^iii^—Ba1—F3^iv^	129.1 (3)	F3^iv^—Ba1—F4^i^	62.5 (4)
F3^i^—Ba1—F1^v^	105.4 (4)	F1^v^—Ba1—F4^i^	54.6 (4)
F3^ii^—Ba1—F1^v^	67.9 (4)	F1^vi^—Ba1—F4^i^	128.6 (4)
F3^iii^—Ba1—F1^v^	125.4 (4)	F1^vii^—Ba1—F4^i^	63.2 (4)
F3^iv^—Ba1—F1^v^	62.1 (4)	F1—Ba1—F4^i^	114.0 (4)
F3^i^—Ba1—F1^vi^	125.4 (4)	F4^ii^—Ba1—F4^i^	117.4 (3)
F3^ii^—Ba1—F1^vi^	105.4 (4)	F4^iv^—Ba1—F4^i^	94.6 (6)
F3^iii^—Ba1—F1^vi^	62.1 (4)	F4^iii^—Ba1—F4^i^	117.4 (3)
F3^iv^—Ba1—F1^vi^	67.9 (4)	F2—Br1—F3	92.6 (6)
F1^v^—Ba1—F1^vi^	90.93 (7)	F2—Br1—F4	177.3 (6)
F3^i^—Ba1—F1^vii^	67.9 (4)	F3—Br1—F4	84.9 (6)
F3^ii^—Ba1—F1^vii^	62.1 (4)	F2—Br1—F1	92.9 (6)
F3^iii^—Ba1—F1^vii^	105.4 (4)	F3—Br1—F1	174.4 (6)
F3^iv^—Ba1—F1^vii^	125.4 (4)	F4—Br1—F1	89.6 (6)
F1^v^—Ba1—F1^vii^	90.93 (7)	Br1—F1—Ba1	132.3 (7)
F1^vi^—Ba1—F1^vii^	165.4 (5)	F2^viii^—Ba2—F2^ix^	136.3 (4)
F3^i^—Ba1—F1	62.1 (4)	F2^viii^—Ba2—F2^x^	63.5 (6)
F3^ii^—Ba1—F1	125.4 (4)	F2^ix^—Ba2—F2^x^	136.3 (4)
F3^iii^—Ba1—F1	67.9 (4)	F2^viii^—Ba2—F2	136.3 (4)
F3^iv^—Ba1—F1	105.4 (4)	F2^ix^—Ba2—F2	63.5 (6)
F1^v^—Ba1—F1	165.4 (5)	F2^x^—Ba2—F2	136.3 (4)
F1^vi^—Ba1—F1	90.93 (7)	F2^viii^—Ba2—F4^xi^	114.0 (5)
F1^vii^—Ba1—F1	90.93 (7)	F2^ix^—Ba2—F4^xi^	109.7 (5)
F3^i^—Ba1—F4^ii^	168.1 (4)	F2^x^—Ba2—F4^xi^	67.9 (5)
F3^ii^—Ba1—F4^ii^	52.0 (4)	F2—Ba2—F4^xi^	68.4 (5)
F3^iii^—Ba1—F4^ii^	62.5 (4)	F2^viii^—Ba2—F4^vii^	67.9 (5)
F3^iv^—Ba1—F4^ii^	95.7 (4)	F2^ix^—Ba2—F4^vii^	68.4 (5)
F1^v^—Ba1—F4^ii^	63.2 (4)	F2^x^—Ba2—F4^vii^	114.0 (5)
F1^vi^—Ba1—F4^ii^	54.6 (4)	F2—Ba2—F4^vii^	109.7 (5)
F1^vii^—Ba1—F4^ii^	114.0 (4)	F4^xi^—Ba2—F4^vii^	178.0 (7)
F1—Ba1—F4^ii^	128.6 (4)	F2^viii^—Ba2—F4^iii^	68.4 (5)
F3^i^—Ba1—F4^iv^	62.5 (4)	F2^ix^—Ba2—F4^iii^	114.0 (5)
F3^ii^—Ba1—F4^iv^	168.1 (4)	F2^x^—Ba2—F4^iii^	109.7 (5)
F3^iii^—Ba1—F4^iv^	95.7 (4)	F2—Ba2—F4^iii^	67.9 (5)
F3^iv^—Ba1—F4^iv^	52.0 (4)	F4^xi^—Ba2—F4^iii^	90.018 (16)
F1^v^—Ba1—F4^iv^	114.0 (4)	F4^vii^—Ba2—F4^iii^	90.018 (15)
F1^vi^—Ba1—F4^iv^	63.2 (4)	F2^viii^—Ba2—F4^xii^	109.7 (5)
F1^vii^—Ba1—F4^iv^	128.6 (4)	F2^ix^—Ba2—F4^xii^	67.9 (5)
F1—Ba1—F4^iv^	54.6 (4)	F2^x^—Ba2—F4^xii^	68.4 (5)
F4^ii^—Ba1—F4^iv^	117.4 (3)	F2—Ba2—F4^xii^	114.0 (5)
F3^i^—Ba1—F4^iii^	95.7 (4)	F4^xi^—Ba2—F4^xii^	90.018 (15)
F3^ii^—Ba1—F4^iii^	62.5 (4)	F4^vii^—Ba2—F4^xii^	90.018 (15)
F3^iii^—Ba1—F4^iii^	52.0 (4)	F4^iii^—Ba2—F4^xii^	178.0 (7)
F3^iv^—Ba1—F4^iii^	168.1 (4)	Br1—F2—Ba2	146.6 (8)
F1^v^—Ba1—F4^iii^	128.6 (4)	Br1—F3—Ba1^xiii^	114.9 (6)
F1^vi^—Ba1—F4^iii^	114.0 (4)	Br1—F4—Ba2^xiv^	120.5 (6)
F1^vii^—Ba1—F4^iii^	54.6 (4)	Br1—F4—Ba1^xiii^	103.2 (5)
F1—Ba1—F4^iii^	63.2 (4)	Ba2^xiv^—F4—Ba1^xiii^	130.1 (5)
			
F3—Br1—F2—Ba2	103.8 (13)	F2—Br1—F3—Ba1^xiii^	158.2 (7)
F1—Br1—F2—Ba2	−75.4 (13)	F4—Br1—F3—Ba1^xiii^	−20.9 (7)

Symmetry codes: (i) *y*−1/2, −*x*+3/2, −*z*+3/2; (ii) *x*−1/2, *y*−1/2, *z*−1/2; (iii) −*x*+3/2, −*y*+3/2, *z*−1/2; (iv) −*y*+3/2, *x*−1/2, −*z*+3/2; (v) −*x*+1, −*y*+1, *z*; (vi) *y*, −*x*+1, −*z*+1; (vii) −*y*+1, *x*, −*z*+1; (viii) *y*−1/2, −*x*+3/2, −*z*+1/2; (ix) −*x*+1, −*y*+2, *z*; (x) −*y*+3/2, *x*+1/2, −*z*+1/2; (xi) *y*, −*x*+2, −*z*+1; (xii) *x*−1/2, *y*+1/2, *z*−1/2; (xiii) *x*+1/2, *y*+1/2, *z*+1/2; (xiv) *x*+1/2, *y*−1/2, *z*+1/2.
