# Supplementary material for: Barium bis­[tetra­fluorido­bromate(III)]
Source: IUCrdata. 2021 Jul 20;6(Pt 7):x210735. doi: 10.1107/S2414314621007355 (PMC9462354; doi:10.1107/S2414314621007355)
Supplement: Supplementary file 3 [file x-06-x210735-sup3.pdf]

Refinement of the crystal structure of barium tetrafluoridobromate(III) from single-crystal X-ray diffraction data

Sergei I. Ivlev and Florian Kraus*

Philipps-Universitaet Marburg, Fachbereich Chemie, Hans-Meerwein-Str. 4, 35032 Marburg, Germany

Correspondence email: f.kraus@uni-marburg.de

Abstract

Single-crystals of barium tetrafluoridobromate(III), $Ba[BrF_4]_2$, were obtained in form of tiny blocks. Crystal structure refinement of $Ba[BrF_4]_2$ from single-crystal X-ray diffraction data confirmed the previous model on basis of powder data [Ivlev *et al.* (2014). *Eur. J. Inorg. Chem.*, pp 6261–6267], but with all atoms refined with anisotropic displacement parameters. The crystal structure consists of two symmetry-independent barium cations that are coordinated by twelve fluorine atoms each, forming edge-sharing polyhedra, and an almost square planar $[BrF_4]^-$ anion. The compound crystallizes in the $Ba[AuF_4]_2$ structure type.

Structure description

The first synthesis of $Ba[BrF_4]_2$ was perfomed by Sharpe & Emeléus (1948) by treating anhydrous barium chloride or barium fluoride with bromine trifluoride. The product was, however, only characterized by means of a quantitative elemental analysis. Thermal properties of $Ba[BrF_4]_2$ were later studied ~~later~~ by Kiselev and co-workers, who investigated the thermal decomposition of $Ba[BrF_4]_2$ to yield barium fluoride (Kiselev *et al.*, 1987). To the best of our knowledge, our report on the crystal structure of $Ba[BrF_4]_2$ determined from X-ray and neutron powder diffraction data at 300 K was the first structural investigation of the title compound (Ivlev *et al.*, 2014). We showed that $Ba[BrF_4]_2$ crystallizes in the space group $I\bar{4}$ and adopts the $Ba[AuF_4]_2$ structure type. Here we present our results on the re-refinement of the crystal structure of $Ba[BrF_4]_2$ from single-crystal X-ray diffraction data at 100 K.

As expected, the ~~unit cell~~ parameters of the single-crystal study at 100 K (Table 1) are smaller than those determined during the powder study at 300 K, a = 9.65081 (11), c = 8.03453 (13) Å, V = 748.32 (2) Å^3 (Ivlev *et al.*, 2014). The crystal structure contains two symmetry-independent Ba^{2+} cations on special Wyckoff position 2a (site symmetry $\bar{4}$..) and 2d ($\bar{4}$..), respectively. Each Ba site is coordinated by twelve F atoms forming edge-sharing polyhedra. The Ba⋯F distances lie in the range of 2.680 (14)⋯~~3.034 (14)~~ Å (powder data at RT yielded the range of 2.696 (3)⋯~~2.81832 (5)~~ Å). The bromine atom occupies the general Wyckoff position 8g and is coordinated by four fluorine atoms also located on general positions in an almost square-planar shape. The resulting Br—F bond lengths are 1.829 (13), 1.861 (12), 1.934 (13), and 1.935 (13) Å, which is comparable with our previous model on basis of powder data (*cf.*: 1.800 (4), 1.856 (4), 1.902 (4), 1.935 (2) Å). The two longer Br—F bond lengths correspond to the F atoms coordinating two barium cations each. The two other fluorine atoms coordinate only to one barium cation each and thus have shorter Br—F bond lengths. The F—Br—F *cis*-angles equal 84.9 (6), 89.6 (6), 92.6 (6), and 92.9 (6)°, which corresponds to the previously published results: 85.14 (16), 90.02 (13), 91.80 (15), 93.04 (18)°. Fig. 1 shows the closest contacts between one $[BrF_4]^-$ anion and its surrounding Ba^{2+} cations**, and Fig. 2 shows the packing of the cations and anions in the crystal structure.**

Synthesis and crystallization

Tiny crystals of barium tetrafluoridobromate(III) were obtained by direct reaction of bromine trifluoride with barium fluoride in a closed Teflon vessel. In contrast to $Rb[BrF_4]$ (Malin *et al.*, 2019) and $Cs[BrF_4]$ (Malin *et al.*, 2020), it was not possible to improve the crystal quality by melting and recrystallization since $Ba[BrF_4]_2$ decomposes before reaching

its melting point.

Refinement details

Details of data collection and structure refinement are given in Table 1. Due to very small size of the crystals, we had to employ a diffractometer with a Cu source to improve the reflection intensities at the cost of a more complicated absorption correction.

Table 1

Experimental details

Crystal data	
Chemical formula	$Ba^{2+}\cdot(BrF_4^-)_2$
M_r	449.16
Crystal system, space group	Tetragonal, $I\bar{4}$
Temperature (K)	100
a, c (Å)	9.5823 (6), 8.0380 (11)
V (Å^3)	738.05 (14)
Z	4
Radiation type	Cu $K\alpha$
μ (mm^{-1})	55.60
Crystal size (mm)	0.02 × 0.02 × 0.01
Data collection	
Diffractometer	Stoe Stadivari
Absorption correction	Multi-scan (*X-AREA* (Stoe, 2020) based on Koziskova *et al.*, (2016))
T_{min}, T_{max}	0.068, 0.362
No. of measured, independent and observed [$I > 2\sigma(I)$] reflections	2012, 746, 688
R_{int}	0.038
$(\sin \theta/\lambda)_{max}$ (Å^{-1})	0.637
Refinement	
$R[F^2 > 2\sigma(F^2)]$, $wR(F^2)$, S	0.049, 0.127, 1.08
No. of reflections	746
No. of parameters	50
$\Delta\rho_{max}$, $\Delta\rho_{min}$ (e Å^{-3})	1.84, −1.05
Absolute structure	Flack x determined using 266 quotients [(I+)-(I-)]/[(I+)+(I-)] (Parsons *et al.*, 2013).
Absolute structure parameter	−0.012 (17)

Computer programs: *X-AREA* (Stoe, 2020), *X-AREA* (Stoe, 2020), *SHELXT* (Sheldrick, 2015a), *SHELXL* (Sheldrick, 2015b), *DIAMOND* (Brandenburg, 2020), *publCIF* (Westrip, 2015).

Acknowledgements

We thank the Deutsche Forschungsgemeinschaft for generous funding.

Funding information

References

Brandenburg, K. (2020). *DIAMOND*. Crystal Impact GbR, Bonn, Germany.

Ivlev, S., Sobolev, V., Hoelzel, M., Karttunen, A. J., Müller, T., Gerin, I., Ostvald, R. & Kraus, F. (2014). *Eur. J. Inorg. Chem.*, pp. 6261–6267.

Kiselev, N. I., Lapshin, O. N., Sadikova, A. T., Sukhoverkhov, V. F. & Churbanov, M. F. (1987). *Visok. Veschestva*, **3**, 178–182.

Koziskova, J., Hahn, F., Richter, J. & Kozisek, J. (2016). *Acta Chim. Slov.* 9, 136–140.

Malin, A. V., Ivlev, S. I., Ostvald, R. V. & Kraus, F. (2019). *IUCrData*, **4**, x191595.

Malin, A. V., Ivlev, S. I., Ostvald, R. V. & Kraus, F. (2020). *IUCrData*, **5**, x200114.

Parsons, S., Flack, H. D. & Wagner, T. (2013). *Acta Cryst*. B69, 249–259.

Sharpe, A. G. & Emeléus, H. J. (1948). *J. Chem. Soc.*, pp. 2135–2138.

Sheldrick, G. M. (2015*a*). *Acta Cryst.* A**71**, 3–8.

Sheldrick, G. M. (2015*b*). *Acta Cryst*. C**71**, 3–8.

Stoe (2020). *X-AREA*. Stoe & Cie GmbH, Darmstadt, Germany.

Westrip, S. P. (2010). *J. Appl. Cryst.* **43**, 920–925.

Figure 1

~~**Caption 1 (including probabilty level and symmetry codes) Caption 2**~~

full crystallographic data

Refinement of the crystal structure of barium tetrafluoridobromate(III) from single-crystal X-ray diffraction data

(I)

Crystal data

$Ba^{2+}\cdot(BrF_4^-)_2$
M_r = 449.16
Tetragonal, $I\bar{4}$
a = 9.5823 (6) Å
c = 8.0380 (11) Å
V = 738.05 (14) Å^3
Z = 4
F(000) = 792
D_x = 4.042 Mg m^{-3}
Cu $K\alpha$ radiation, λ = 1.54186 Å
Cell parameters from 2706 reflections
θ = 6.5–79.3°
μ = 55.60 mm^{-1}
T = 100 K
Block, colorless
0.02 × 0.02 × 0.01 mm

Data collection

Stoe Stadivari diffractometer
Radiation source: GeniX 3D HF Cu
Graded multilayer mirror monochromator
Detector resolution: 5.81 pixels mm^{-1}
rotation method, ω scans
Absorption correction: multi-scan (*X-AREA* (Stoe, 2020) based on Koziskova *et al.*, (2016))
T_{min} = 0.068, T_{max} = 0.362
2012 measured reflections
746 independent reflections
688 reflections with $I > 2\sigma(I)$
R_{int} = 0.038
θ_{max} = 79.1°, θ_{min} = 6.5°
$h = -11 \rightarrow 12$
$k = -10 \rightarrow 6$
$l = -10 \rightarrow 9$

Refinement

Refinement on F^2
Least-squares matrix: full
$R[F^2 > 2\sigma(F^2)]$ = 0.049
$wR(F^2)$ = 0.127
S = 1.08
746 reflections
50 parameters
0 restraints
Primary atom site location: structure-invariant direct methods
$w = 1/[\sigma^2(F_o^2) + (0.091P)^2 + 5.3796P]$ where $P = (F_o^2 + 2F_c^2)/3$
$(\Delta/\sigma)_{max}$ < 0.001
$\Delta\rho_{max}$ = 1.84 e Å^{-3}
$\Delta\rho_{min}$ = −1.05 e Å^{-3}
Absolute structure: Flack x determined using 266 quotients [(I+)-(I-)]/[(I+)+(I-)] (Parsons *et al.*, 2013).
Absolute structure parameter: −0.012 (17)

Special details

Geometry. All e.s.d.'s (except the e.s.d. in the dihedral angle between two l.s. planes) are estimated using the full covariance matrix. The cell e.s.d.'s are taken into account individually in the estimation of e.s.d.'s in distances, angles and torsion angles; correlations between e.s.d.'s in cell parameters are only used when they are defined by crystal symmetry. An approximate (isotropic) treatment of cell e.s.d.'s is used for estimating e.s.d.'s involving l.s. planes.

Fractional atomic coordinates and isotropic or equivalent isotropic displacement parameters (Å^2)

	x	y	z	U_{iso}*/U_{eq}
Ba1	0.500000	0.500000	0.500000	0.0243 (6)
Br1	0.77576 (19)	0.84257 (18)	0.6325 (2)	0.0238 (5)
F1	0.7435 (16)	0.6574 (14)	0.5444 (17)	0.041 (3)

Ba2	0.500000	1.000000	0.250000	0.0293 (6)
F2	0.6192 (14)	0.9136 (15)	0.5334 (19)	0.041 (3)
F3	0.8236 (13)	1.0143 (13)	0.7242 (17)	0.036 (3)
F4	0.9413 (13)	0.7748 (13)	0.744 (2)	0.040 (3)

Atomic displacement parameters (Å^2)

	U^{11}	U^{22}	U^{33}	U^{12}	U^{13}	U^{23}
Ba1	0.0219 (7)	0.0219 (7)	0.0291 (12)	0.000	0.000	0.000
Br1	0.0251 (10)	0.0197 (9)	0.0267 (9)	−0.0021 (7)	−0.0004 (8)	0.0000 (7)
F1	0.050 (8)	0.030 (7)	0.043 (7)	−0.013 (6)	0.008 (6)	−0.008 (5)
Ba2	0.0257 (8)	0.0257 (8)	0.0364 (13)	0.000	0.000	0.000
F2	0.033 (7)	0.042 (7)	0.048 (8)	0.004 (6)	−0.009 (6)	−0.004 (6)
F3	0.042 (7)	0.027 (6)	0.038 (8)	−0.001 (5)	−0.006 (6)	−0.003 (5)
F4	0.034 (7)	0.029 (6)	0.057 (8)	0.003 (6)	−0.017 (7)	−0.006 (6)

Geometric parameters (Å, º)

Ba1—F3^{i}	2.791 (13)	Br1—F2	1.829 (13)
Ba1—F3^{ii}	2.791 (13)	Br1—F3	1.861 (12)
Ba1—F3^{iii}	2.791 (13)	Br1—F4	1.934 (13)
Ba1—F3^{iv}	2.791 (13)	Br1—F1	1.935 (13)
Ba1—F1^{v}	2.801 (14)	Ba2—F2^{viii}	2.680 (14)
Ba1—F1^{vi}	2.801 (14)	Ba2—F2^{ix}	2.680 (14)
Ba1—F1^{vii}	2.801 (14)	Ba2—F2^{x}	2.680 (14)
Ba1—F1	2.801 (14)	Ba2—F2	2.680 (14)
Ba1—F4^{ii}	3.034 (14)	Ba2—F4^{xi}	2.693 (12)
Ba1—F4^{iv}	3.034 (14)	Ba2—F4^{vii}	2.693 (12)
Ba1—F4^{iii}	3.034 (14)	Ba2—F4^{iii}	2.693 (12)
Ba1—F4^{i}	3.034 (14)	Ba2—F4^{xii}	2.693 (12)
F3^{i}—Ba1—F3^{ii}	129.1 (3)	F4^{ii}—Ba1—F4^{iii}	94.6 (6)
F3^{i}—Ba1—F3^{iii}	129.1 (3)	F4^{iv}—Ba1—F4^{iii}	117.4 (3)
F3^{ii}—Ba1—F3^{iii}	74.9 (6)	F3^{i}—Ba1—F4^{i}	52.0 (4)
F3^{i}—Ba1—F3^{iv}	74.9 (6)	F3^{ii}—Ba1—F4^{i}	95.7 (4)
F3^{ii}—Ba1—F3^{iv}	129.1 (3)	F3^{iii}—Ba1—F4^{i}	168.1 (4)
F3^{iii}—Ba1—F3^{iv}	129.1 (3)	F3^{iv}—Ba1—F4^{i}	62.5 (4)
F3^{i}—Ba1—F1^{v}	105.4 (4)	F1^{v}—Ba1—F4^{i}	54.6 (4)
F3^{ii}—Ba1—F1^{v}	67.9 (4)	F1^{vi}—Ba1—F4^{i}	128.6 (4)
F3^{iii}—Ba1—F1^{v}	125.4 (4)	F1^{vii}—Ba1—F4^{i}	63.2 (4)
F3^{iv}—Ba1—F1^{v}	62.1 (4)	F1—Ba1—F4^{i}	114.0 (4)
F3^{i}—Ba1—F1^{vi}	125.4 (4)	F4^{ii}—Ba1—F4^{i}	117.4 (3)
F3^{ii}—Ba1—F1^{vi}	105.4 (4)	F4^{iv}—Ba1—F4^{i}	94.6 (6)
F3^{iii}—Ba1—F1^{vi}	62.1 (4)	F4^{iii}—Ba1—F4^{i}	117.4 (3)
F3^{iv}—Ba1—F1^{vi}	67.9 (4)	F2—Br1—F3	92.6 (6)
F1^{v}—Ba1—F1^{vi}	90.93 (7)	F2—Br1—F4	177.3 (6)
F3^{i}—Ba1—F1^{vii}	67.9 (4)	F3—Br1—F4	84.9 (6)
F3^{ii}—Ba1—F1^{vii}	62.1 (4)	F2—Br1—F1	92.9 (6)
F3^{iii}—Ba1—F1^{vii}	105.4 (4)	F3—Br1—F1	174.4 (6)
F3^{iv}—Ba1—F1^{vii}	125.4 (4)	F4—Br1—F1	89.6 (6)
F1^{v}—Ba1—F1^{vii}	90.93 (7)	Br1—F1—Ba1	132.3 (7)
F1^{vi}—Ba1—F1^{vii}	165.4 (5)	F2^{viii}—Ba2—F2^{ix}	136.3 (4)

F3^{i}—Ba1—F1	62.1 (4)	F2^{viii}—Ba2—F2^{x}	63.5 (6)
F3^{ii}—Ba1—F1	125.4 (4)	F2^{ix}—Ba2—F2^{x}	136.3 (4)
F3^{iii}—Ba1—F1	67.9 (4)	F2^{viii}—Ba2—F2	136.3 (4)
F3^{iv}—Ba1—F1	105.4 (4)	F2^{ix}—Ba2—F2	63.5 (6)
F1^{v}—Ba1—F1	165.4 (5)	F2^{x}—Ba2—F2	136.3 (4)
F1^{vi}—Ba1—F1	90.93 (7)	F2^{viii}—Ba2—F4^{xi}	114.0 (5)
F1^{vii}—Ba1—F1	90.93 (7)	F2^{ix}—Ba2—F4^{xi}	109.7 (5)
F3^{i}—Ba1—F4^{ii}	168.1 (4)	F2^{x}—Ba2—F4^{xi}	67.9 (5)
F3^{ii}—Ba1—F4^{ii}	52.0 (4)	F2—Ba2—F4^{xi}	68.4 (5)
F3^{iii}—Ba1—F4^{ii}	62.5 (4)	F2^{viii}—Ba2—F4^{vii}	67.9 (5)
F3^{iv}—Ba1—F4^{ii}	95.7 (4)	F2^{ix}—Ba2—F4^{vii}	68.4 (5)
F1^{v}—Ba1—F4^{ii}	63.2 (4)	F2^{x}—Ba2—F4^{vii}	114.0 (5)
F1^{vi}—Ba1—F4^{ii}	54.6 (4)	F2—Ba2—F4^{vii}	109.7 (5)
F1^{vii}—Ba1—F4^{ii}	114.0 (4)	F4^{xi}—Ba2—F4^{vii}	178.0 (7)
F1—Ba1—F4^{ii}	128.6 (4)	F2^{viii}—Ba2—F4^{iii}	68.4 (5)
F3^{i}—Ba1—F4^{iv}	62.5 (4)	F2^{ix}—Ba2—F4^{iii}	114.0 (5)
F3^{ii}—Ba1—F4^{iv}	168.1 (4)	F2^{x}—Ba2—F4^{iii}	109.7 (5)
F3^{iii}—Ba1—F4^{iv}	95.7 (4)	F2—Ba2—F4^{iii}	67.9 (5)
F3^{iv}—Ba1—F4^{iv}	52.0 (4)	F4^{xi}—Ba2—F4^{iii}	90.018 (16)
F1^{v}—Ba1—F4^{iv}	114.0 (4)	F4^{vii}—Ba2—F4^{iii}	90.018 (15)
F1^{vi}—Ba1—F4^{iv}	63.2 (4)	F2^{viii}—Ba2—F4^{xii}	109.7 (5)
F1^{vii}—Ba1—F4^{iv}	128.6 (4)	F2^{ix}—Ba2—F4^{xii}	67.9 (5)
F1—Ba1—F4^{iv}	54.6 (4)	F2^{x}—Ba2—F4^{xii}	68.4 (5)
F4^{ii}—Ba1—F4^{iv}	117.4 (3)	F2—Ba2—F4^{xii}	114.0 (5)
F3^{i}—Ba1—F4^{iii}	95.7 (4)	F4^{xi}—Ba2—F4^{xii}	90.018 (15)
F3^{ii}—Ba1—F4^{iii}	62.5 (4)	F4^{vii}—Ba2—F4^{xii}	90.018 (15)
F3^{iii}—Ba1—F4^{iii}	52.0 (4)	F4^{iii}—Ba2—F4^{xii}	178.0 (7)
F3^{iv}—Ba1—F4^{iii}	168.1 (4)	Br1—F2—Ba2	146.6 (8)
F1^{v}—Ba1—F4^{iii}	128.6 (4)	Br1—F3—Ba1^{xiii}	114.9 (6)
F1^{vi}—Ba1—F4^{iii}	114.0 (4)	Br1—F4—Ba2^{xiv}	120.5 (6)
F1^{vii}—Ba1—F4^{iii}	54.6 (4)	Br1—F4—Ba1^{xiii}	103.2 (5)
F1—Ba1—F4^{iii}	63.2 (4)	Ba2^{xiv}—F4—Ba1^{xiii}	130.1 (5)

F3—Br1—F2—Ba2	103.8 (13)	F2—Br1—F3—Ba1^{xiii}	158.2 (7)
F1—Br1—F2—Ba2	−75.4 (13)	F4—Br1—F3—Ba1^{xiii}	−20.9 (7)

Symmetry codes: (i) *y*−1/2, −*x*+3/2, −*z*+3/2; (ii) *x*−1/2, *y*−1/2, *z*−1/2; (iii) −*x*+3/2, −*y*+3/2, *z*−1/2; (iv) −*y*+3/2, *x*−1/2, −*z*+3/2; (v) −*x*+1, −*y*+1, *z*; (vi) *y*, −*x*+1, −*z*+1; (vii) −*y*+1, *x*, −*z*+1; (viii) *y*−1/2, −*x*+3/2, −*z*+1/2; (ix) −*x*+1, −*y*+2, *z*; (x) −*y*+3/2, *x*+1/2, −*z*+1/2; (xi) *y*, −*x*+2, −*z*+1; (xii) *x*−1/2, *y*+1/2, *z*−1/2; (xiii) *x*+1/2, *y*+1/2, *z*+1/2; (xiv) *x*+1/2, *y*−1/2, *z*+1/2.
